# Significance of CD103^+^ tissue-resident memory T cells for predicting the effectiveness of immune checkpoint inhibitors in esophageal cancer

**DOI:** 10.1186/s12885-023-11438-5

**Published:** 2023-10-20

**Authors:** Seiji Natsuki, Hiroaki Tanaka, Masaki Nishiyama, Sota Deguchi, Yuichiro Miki, Mami Yoshii, Tatsuro Tamura, Takahiro Toyokawa, Shigeru Lee, Kiyoshi Maeda

**Affiliations:** 1 Department of Gastroenterological Surgery, Osaka City University Graduate School of Medicine, 1-4-3 Asahi-Machi, Abeno-Ku, Osaka, 545-8585 Japan; 2 Department of Gastroenterological Surgery, Fuchu Hospital, Osaka, Japan

**Keywords:** CD103, Esophageal cancer, Tissue-resident memory T cell, Immune checkpoint inhibitor, Antitumor immunity, PD-L1

## Abstract

**Background:**

Immune checkpoint inhibitors (ICIs), including nivolumab, have been approved to treat esophageal cancer. However, these remedies are not fit for all patients with esophageal cancer; therefore, a predictive surrogate marker is needed to assess their effectiveness. CD103^+^CD8^+^ tumor-infiltrating lymphocytes, defined as tissue-resident memory T cells (T_RM_), are promising indicators of response to ICIs, but it remains to be elucidated. This study investigated the association between the efficacy of ICIs and T_RM_.

**Methods:**

The relationships between T_RM_ infiltrating esophageal cancer, clinicopathological features, and prognosis after nivolumab initiation were examined using immunostaining. Tissue samples were obtained from surgically resected specimens of 37 patients with esophageal cancer who received nivolumab as a secondary or subsequent therapy. In addition, T_RM_ infiltration was compared with programmed death-ligand 1 (PD-L1) expression and blood count parameters as predictors of nivolumab effectiveness.

**Results:**

T_RM_-rich patients had a significant survival benefit after nivolumab initiation (12-months overall survival 70.8% vs 37.2%, *p* = 0.0485; 12-months progression-free survival 31.2% vs 0%, *p* = 0.0153) and experienced immune-related adverse events more frequently than T_RM_-poor patients (6 vs 2 patients). T_RM_ infiltration was weakly correlated with PD-L1 positivity (*r* = 0.374, *p* = 0.022), but T_RM_ may indicate more sensitive response to ICIs than PD-L1 expression in this study. Some blood test parameters also weakly correlated with T_RM_ but did not impact prognosis.

**Conclusions:**

T_RM_-rich patients have a favorable prognosis after nivolumab initiation. Our results suggest that T_RM_ are vital for antitumor immunity and are a promising predictor of ICIs effectiveness.

**Supplementary Information:**

The online version contains supplementary material available at 10.1186/s12885-023-11438-5.

## Introduction

Esophageal squamous cell carcinoma (ESCC) has an unfavorable prognosis, and few treatment options exist. The recent emergence of immune checkpoint inhibitors (ICI) for programmed death 1 (PD-1) has led to a significant turning point in ESCC treatment approaches [1]. Nivolumab, an ICI against PD-1, demonstrated survival benefits for patients with unresectable advanced or recurrent esophageal cancer in a phase III trial (ATRACTION-3) [2] and was approved as a second-line treatment option in advanced ESCC. ICIs have been hitting a landmark for ESCC treatment, but not all patients with ESCC necessarily fit ICIs and receive survival benefits. Therefore, various studies have been conducted to identify biomarkers for predicting the effectiveness of ICIs. Currently, programmed death ligand 1 (PD-L1) measurement is a treatment indicator [2, 3]; however, it requires expert inspection and is not easy to perform. In addition, we aim to investigate additional streamlined biomarkers in this study.

Tumor-infiltrating lymphocytes (TILs) are essential in antitumor immunity against various cancers [4]. In ESCC, TILs are also associated with a favorable prognosis [5]; in particular, CD103^+^ TILs, called tissue-resident memory T cells (T_RM_), provide further survival benefits, as shown in our previous report [6]. Previously, T_RM_ has attracted attention as an immunotherapeutic target in other types of cancer [7–10]. However, there are few reports on the clinical relevance of T_RM_ infiltration and ICIs efficacy. This study examined the possibility of using T_RM_ in ESCC as a simpler and more useful biomarker for ICIs effectiveness.

## Methods

### Patients and samples

This study included 71 patients with recurrent esophageal cancer who received nivolumab as a secondary or subsequent therapy at Osaka City University (currently Osaka Metropolitan University; Osaka, Japan) between March 2020 and April 2022. For T_RM_ evaluation, surgical specimens are essential. It is uncertain whether biopsied specimens are appropriate to assess T_RM_ infiltration because biopsy specimens are too small and relatively superficial compared to surgical specimens. Thus, to evaluate the primary tumor specimens, we excluded patients who (i) had never undergone radical esophagectomy (27 patients), (ii) histologically had no SCC (one patient), and (iii) had incomplete data (two patients). Forty-one patients who underwent radical esophagectomy at our department were included in this study. Primary tumor samples were obtained from the 41 included patients, but the primary lesions of four patients could not be evaluated because they were reduced and disappeared almost entirely after the preoperative treatments: therefore, 37 patients were enrolled and analyzed.

Indications for chemotherapy and details of the regimens were done following esophageal cancer treatment guidelines [1]. The preoperative regimen consisted of two cycles of 5-fluorouracil + cisplatin (FP), 5-fluorouracil + nedaplatin (FGP), and docetaxel + cisplatin + 5-fluorouracil (DCF). For recurrent cases, nivolumab or paclitaxel were used in addition to the former regimens.

The regimen schedules have been reported previously [6]. FP consisted of a 4-week cycle of fluorouracil at 800 mg per square meter of body surface area on days 1 through 5 and cisplatin at 80 mg per square meter on day 1. FGP consisted of a 4-week cycle of fluorouracil at 800 mg per square meter on days 1 through 5 and nedaplatin at 90 mg per square meter on day 1. DCF consisted of a 4-week cycle of fluorouracil at 800 mg per square meter on days 1 through 5, cisplatin at 80 mg per square meter on day 1, and docetaxel at 30 mg per square meter on days 1 and 15. In addition, nivolumab was administered every two weeks at 240 mg, and paclitaxel was administered in a 7-week cycle at 100 mg per square meter of body surface area on days 1, 8, 15, 22, 29, and 36.

The chemotherapy dose was reduced as needed in accordance with the patients’ condition and adverse events. Treatment after recurrence was continued until disease progression or unacceptable adverse events occurred. In case patients had a recurrence within six months after surgery or did not fit neoadjuvant chemotherapy due to chemo-induced toxicity, they commonly received treatments except neoadjuvant chemotherapy on recurrence. The response of the target lesions to nivolumab was evaluated by attending physicians according to the Response Evaluation Criteria in Solid Tumors guidelines, version 1.1 [11]. We defined the tumor locations as upper thoracic esophagus /middle thoracic esophagus /lower thoracic esophagus, and histological type as differentiated type (well differentiated/moderately differentiated)/undifferentiated type (poorly differentiated) based on the 12^th^ edition of the Japanese Classification of Esophageal Cancer [1]. All pathological stages were recorded according to the 8^th^ edition of the Union International Cancer Control TNM Classification [12].

This study was conducted following the Declaration of Helsinki and approved by the Osaka City University Ethics Committee. Written informed consent was obtained from all patients before enrollment.

### Immunohistochemistry

Immunohistochemistry was performed as previously described [6]. All immunohistochemical analyses were performed on 4-µm-thick sections from paraffin-embedded primary tumor blocks from ESCC patient specimens. The sections were autoclaved for antigen retrieval using Target Retrieval Solution (100 × citrate buffer [pH 6.0]; Dako, Agilent Technologies, Inc.). Nonspecific binding was blocked using nonspecific staining blocking reagent (prediluted; Nichirei Biosciences, Inc.). The sections were then reacted at 4 °C overnight with the primary antibody as follows: rabbit monoclonal anti-CD103 antibody (clone: EPR4166; cat. no. ab1292202; 1/1000; Abcam), mouse monoclonal anti-CD8 antibody (clone: C8/144B; cat. no. M7103; 1/250; Dako), rabbit monoclonal anti-CD69 antibody (clone: EPR21814; cat. no. ab233396; 1/500; Abcam), rabbit monoclonal anti-TCF1/ TCF7 antibody (clone: C63D9; cat. no. 2203; 1/200; Cell Signaling), rabbit monoclonal anti-PD-L1 antibody (clone: 28–8; cat. no. ab205921; 1/200; Abcam), and mouse monoclonal anti-CD20 antibody (clone: L26: cat. no. IR604; prediluted, Dako). The sections were then incubated with the secondary antibody for 10 min at room temperature. After washing in phosphate-buffered saline, the sections were visualized with 3–3’-diamino-benzidine for 5 min and then counterstained with hematoxylin.

### Assessment of immunohistochemical staining

Primary tumor sections stained with anti-CD103 antibody were scanned at × 400 magnification. Three representative high-power fields containing CD103^+^ cells were randomly selected, and the average number of CD103^+^ cells in the three fields was calculated. The cut-off value was determined from a time-dependent receiver operating characteristic (ROC) curve of 12-month progression-free survival (PFS) after nivolumab initiation as a secondary or subsequent treatment. The ROC curve is shown in Supplementary Fig. 1a. Patients were divided into two groups according to this value. Tumor sections stained with anti-PD-L1 antibody were detected at × 40 magnification and scanned at × 100 magnification. We evaluated the rate of tumor cells displaying membrane PD-L1 staining within tumor cells, called tumor proportional score (TPS). Three fields with the most PD-L1^+^ tumor cells were randomly selected, and the average TPS was calculated. Following the clinical trial, ATTRACTION-3 [2], we classified patients according to the borderlines of TPS: 1%, 5%, or 10%. All microscopic images were imported from the DP-73 digital photo filing system (Olympus).

### Blood test parameters

T_RM_, which is a TIL, is possibly related with the number of lymphocytes in blood tests. Additionally, blood tests parameters were reported to possibly predict the response to nivolumab [13–15]. Therefore, we examined association between T_RM_ infiltration and blood test parameters. We investigated the association between laboratory parameters and CD103^+^ TILs. Laboratory data including complete blood count (neutrophils, lymphocytes, monocytes, and platelets), were collected before and after (postoperative day three) the surgery and before nivolumab initiation. The peripheral blood cells ratios were calculated as follows: lymphocyte-to-monocyte ratio (LMR), Total lymphocyte counts (TLC), neutrophil-to-lymphocyte ratio (NLR), and Platelet-to-lymphocyte ratio (PLR). We examined whether these parameters at each time point were correlated with CD103^+^ TILs and nivolumab-related prognosis.

### Determination and evaluation of the efficacy of nivolumab in target lesions

We assessed the response of tumors to nivolumab following the Response Evaluation Criteria in Solid Tumors guidelines version 1.1 [11] as follows: the best overall response (BOR), the objective response rate (ORR; the percentage of patients whose BOR was either complete response (CR) or partial response (PR)), the disease control rate (DCR; the percentage of patients with CR, PR, or stable disease (SD)), and the duration of response (DOR; time from the first response to the first detected tumor progression or death).

### Statistical analyses

All statistical analyses were performed using EZR, an R software with a modified version of R commander designed to add statistical functions frequently used in biostatistics. Fisher’s exact probability test was used to compare categorical variables. Mann–Whitney *U* test was used to compared continuous variables. Overall survival (OS) and PFS after nivolumab initiation were compared using the Kaplan–Meier method, and the significance of differences in survival was analyzed using the log-rank test. The date of initial nivolumab administration was set as the starting point for OS measurement. PFS is the time from nivolumab initiation to death or recurrent lesion progression. The Cox proportional hazards model was used for univariate and multivariate analyses of the prognostic factors. Correlations between some parameters and CD103 expression were examined using Spearman’s rank correlation coefficient. *P*-values < 0.05 were considered to indicate statistically significant differences.

## Results

### Association of intra-tumoral T_RM_ with clinicopathological characteristics

**Fig. 1:**
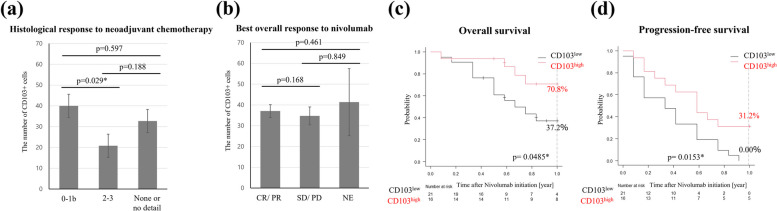
**a**, **b** Bar graphs show the mean ± standard error of the number of CD103 + cells. *p < 0.05, statistically significant. **a** Comparison of the histological response to neoadjuvant chemotherapy. **b** Comparison of the BOR to nivolumab. **c**, **d** Comparison of OS and PFS after nivolumab beginning between two groups using Kaplan–Meier plots and the log-rank test. *p < 0.05, statistically significant

Thiry-seven patients with relevant clinicopathological findings were included in our study (Table 1). The mean patient age was 69 years (range, 40–83 years), and the study group included 27 men (73.0%) and 10 women (27/0%). The mean follow-up time after nivolumab administration was nine months (range, 1–29 months), and the mean duration of nivolumab administration was six months (range, 0–29 months). Based on the average of CD103^+^ infiltrates within the primary tumors, we classified patients into two groups. We used 34.5 cells per field as a cut-off value based on the ROC curve. The values were divided into CD103^high^ and CD103^low^ (Table 2). There were no significant differences in tumor location, histological type, or pathological staging. After recurrence, duration of treatments before nivolumab and treatment contents did not differ significantly between the two groups. Nine patients with recurrent esophageal cancer didn’t receive chemotherapy before nivolumab since they had early recurrence after surgery or did not fit neoadjuvant chemotherapy in chemo-induced toxicity and renal failure. The number of organs with recurrence and recurrent style didn’t observe significant differences between the groups. Although there were no statistical differences, CD103^high^ tended to experience more irAEs than CD103^low^. Eight patients experienced immune-related Adverse Events (irAEs) (one hepatitis, two pneumonia, three hypothyroidism, two skin lesion). Five patients experienced irAEs within six months, and three experienced irAEs after seven months. Additionally, we examined relationships of the number of CD103^+^ cells with the histological response to neoadjuvant chemotherapy (Fig. 1a) and the BOR to nivolumab (Fig. 1b). In neoadjuvant chemotherapy, poor response group (grade 0-1b) had more CD103^+^ cells significantly than good response group (grade 2–3) (*p* = 0.029). In the BOR to nivolumab, patients evaluated as CR/ PR had no significant difference in CD103 expression from those evaluated as SD/ PD (*p* = 0.168).

**Table 1:** Clinicopathological characteristics (*n* = 37)

Variables		N or median (range)
Age		69 (40–83)
Sex	Male/ Female	27/ 10
Location	Ut/ Mt/ Lt	10/ 16/ 11
Histology	well/ mod/por/ scc (unidentified)	2/ 17/ 6/ 12
pT-category^a^	1/2/3/4	9/ 6/ 19/ 3
pN-category^a^	0/1/2/3	11/ 10/ 10/ 6
pN-number		1 (0–12)
pStage^a^	I/ II/ III/ IV	2/ 10/ 14/ 11
NAC regimen	FP or FGP/ DCF/ none	9/ 23/ 5
Histological grade	0-1b/ 2–3/ none or no details	23/ 4/ 10

*scc* squamous cell carcinoma, *well* well-differentiated scc, *mod* moderate-differentiated scc, *por* poorly-differentiated scc, *NAC* neoadjuvant chemotherapy, *FP* fluorouracil and cisplatin, *FGP* fluorouracil and nedaplatin, *DCF* docetaxel, cisplatin and fluorouracil

^a^TNM Classification of Esophageal squamous cell carcinoma, 8th ed

**Table 2:** Association between clinicopathological findings and CD103 expression within primary tumors

		CD103-positive T cells within primary tumors		
Variables	State	Low (*n*=21)	High (*n*=16)	*P*-value†
Age	>=65/ <65	16/ 5	8/ 8	0.165
Sex	Female/ Male	6/ 15	4/ 12	1
Location	Upper/ Middle/ Lower thoracic	6/ 8/ 7	4/ 8/ 4	0.768
Histology	differentiated/ undifferentiated/ unidentified scc	10/ 3/ 8	9/ 3/ 4	0.668
pT-category^a^	1-2/ 3-4	11/ 10	4/ 12	0.176
pN-category^a^	0-1/ 2-3	10/ 11	11/ 5	0.316
pStage^a^	I-II/ III-IV	7/ 14	4/ 12	0.723
NAC regimen	FP or FGP/ DCF/ none	6/ 8/ 2	3/ 15/ 3	0.275
Histological grade	0-1b/ 2-3/ none or no details	8/ 4/ 4	15/ 0/ 6	0.064
Duration of recurrent treatments before nivolumab	median [months] (range)	4.0 (0-90)	1.5 (0-51)	0.405
Radiation for recurrent treatment	present (%)	7 (33.3%)	6 (37.5%)	1
The number of chemotherapies for recurrence before nivolumab	One/ Two/ Three/ None	12/ 3/ 0/ 6	12/ 0/ 1/ 3	0.221
Chemotherapy regimen before nivolumab	FP or FGP/ DCF/ Other/ Multiple	10/ 7/ 1/ 3	9/ 6/ 0/ 1	0.884
The number of recurrent organs	One/ Two/ three	11/6/4	11/4/1	0.464
Lymphatic metastasis	present (%)	17 (81%)	13 (81.3%)	1
hematogenous metastasis	present (%)	9 (42.9%)	6 (37.5%)	1
Local recurrence	present (%)	3 (14.3%)	3 (18.8%)	1
Dissemination	present (%)	5 (23.8%)	0 (0%)	0.057
Best overall response	CR, PR/ SD, PD/ NE	2/ 16/ 3	6/ 9/ 1	0.118
Objective response rate		11.1%	40.0%	0.101
Disease control rate		44.4%	73.3%	0.158
Duration of response	median [months] (range)	2.0 (1.0-13)	7.0 (1.0-29)	0.017*
irAE	present (%)	2 (9.5%)	6 (37.5%)	0.055
	hepatits	0	1	
	interstinal pneumonia	0	2	
	hypothyroidism	1	2	
	skin lesion	1	1	

*SCC* Squamous Cell Carcinoma, *NAC* Neoadjuvant chemothrapy, *FP* fluorouracil and cisplatin, *FGP* fluorouracil and nedaplatin, *DCF* docetaxel, cisplatin and fluorouracil, *CR* complete response, *PR* partial response, *SD* stable disease, *PD* progressive disease, *NE* not evaluable, *irAE* immune related adverse events

^†^Fisher's probability exact test for categorical variables. Mann–Whitney *U* test for continuous variables. *statistically significant; *p* < 0.05

^a^TNM Classification of Esophageal squamous cell carcinoma, 8th ed

### CD103^+^ cells were associated with favorable survival after nivolumab start

We investigated the association between CD103^+^ cell infiltration and patients prognosis (Fig. 1c, d). In both OS and PFS, CD103^high^ was associated with a better prognosis than CD103^low^. The 12-month OS rate was 70.8% versus 37.2%, and the 12-month PFS rate was 31.2% versus 0% in the CD103^high^ and CD103^low^, respectively.

**Fig. 2:**
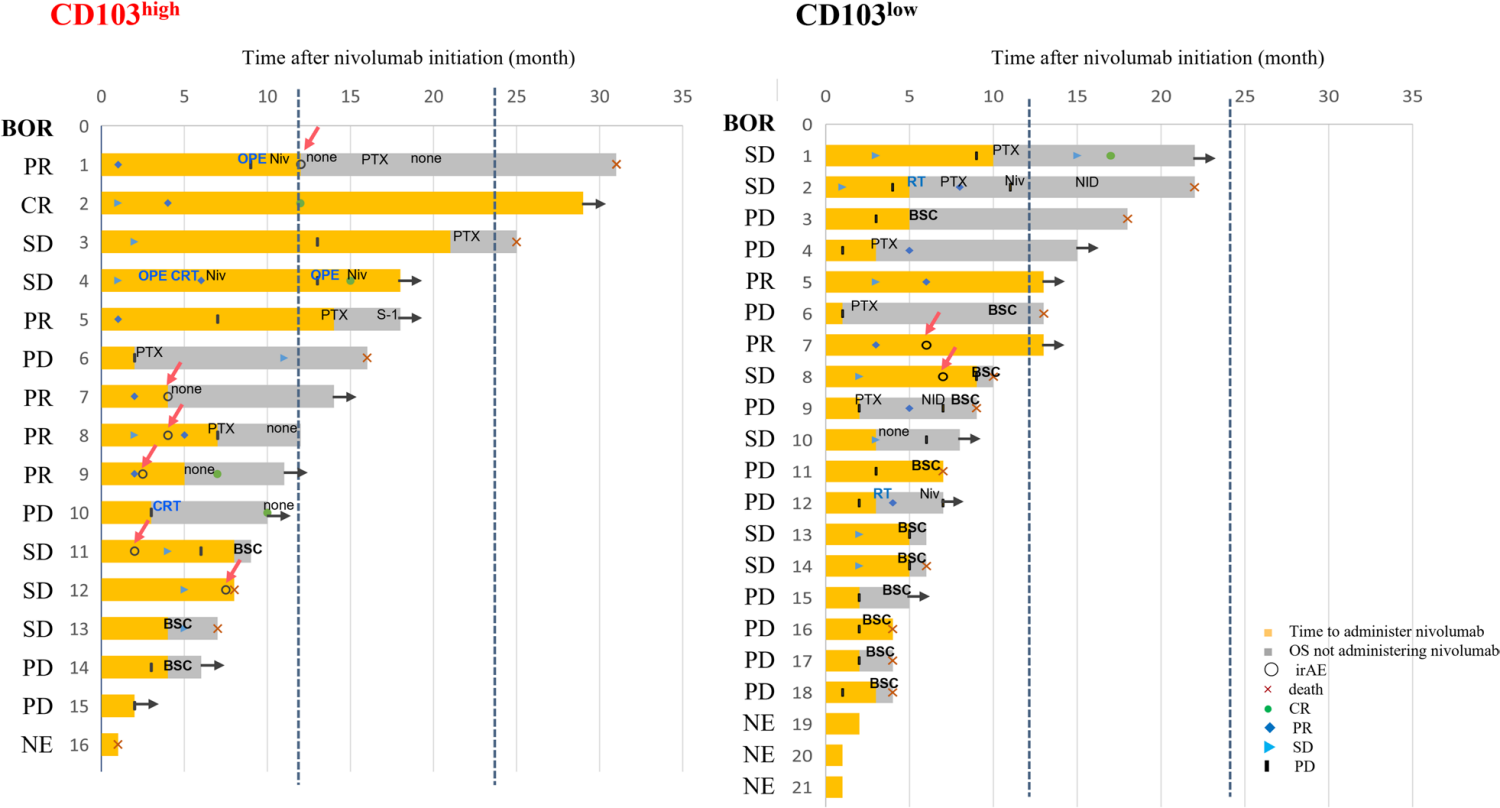
Swimmer plot. We compared the treatment time courses between CD103^high^ and CD103^low^. BOR, Best Overall Response; CR, complete response; PR, partial response; SD, stable disease; PD, progressive disease; NE, not evaluable; irAE, immune-related Adverse Events; BSC, best supportive care; Niv, nivolumab; PTX, paclitaxel; S-1, tegaful gimeracil oteracil potassium; NID, new investigational drug; OPE, operative surgery; CRT, chemoradiotherapy; RT, radiotherapy

The treatment time courses for both groups were organized by drawing a swimmer plot (Fig. 2). Of course, there were differences in survival between the two groups, and fewer patients in CD103^high^ discontinued any remedies and transitioned to best-supported care than their counterparts in CD103^low^. CD103^high^ experienced relatively more irAEs. In CD103^high^ and CD103^low^ groups, respectively, the ORR was 6/15 (40.0%) versus 2/18 (11.1%), the DCR was 11/15 (73.3%) versus 8/18 (44.4%), the median DOR was 7.0 months (range, 1.0–29 months) versus 2.0 months (range, 1.0–13 months) (Table 2). The two groups had no statistical differences in the ORR (*p* = 0.101) and the DCR (*p* = 0.158), but the DOR differed significantly between the two groups (*p* = 0.017).

### Prognostic factors

Using the Cox proportional hazards model, we performed univariate and multivariate analyses of prognostic factors after the commencement of nivolumab (Table 3). CD103^+^ cell infiltration was a dependent prognostic factor like the size of the recurrent lesions. In this study, laboratory data and PD-L1 expression were not significant prognostic factors.

**Table 3:** Univariate and multivariate analyses of prognostic factors after Nivolumab administration in reccurrent ESCC patients

	12-month Overall Survival afterNivolumab administration					
	Univariate analysis			Multivariate analysis		
Variables	Hazard ratio	(95% CI)	*P*-value†	Hazard ratio	(95% CI)	*P*-value†
Age (< 65, > = 65)	0.644	(0.255–1.624)	0.351			
Sex (Female/ Male)	1.569	(0.515–4.781)	0.428			
Histology (differentiated/ undifferentiated scc)	1.130	(0.310–4.115)	0.853			
Histological grade (0–1/ 2–3)	0.481	(0.109–2.119)	0.331			
Neoadjuvant chemotherapy (FP or FGP/ DCF)	0.523	(0.203–1.350)	0.180			
irAE (absent/ presnt)	0.509	(0.147–1.765)	0.287			
pT-category (T1-2/ T3-4)^a^	1.034	(0.387–2.766)	0.947			
pN-category (N0-1/ 2–3)^a^	0.491	(0.175–1.379)	0.177			
pStage (II/ III-IV)^a^	0.576	(0.351–0.945)	0.029*	0.609	(0.356–1.043)	0.071
The size of recurrent lesions (< 35, > = 35)	6.207	(2.138–18.02)	< 0.001*	13.400	(3.034–59.17)	< 0.001*
Local recurrence (absent/ present)	1.234	(0.357–4.269)	0.740			
Lympatic metastasis (absent/ present)	0.795	(0.283–2.236)	0.664			
Hematogenous metastatis (absent/ present)	1.599	(0.629–4.065)	0.324			
Hepatic metastasis (absent/ present)	1.910	(0.546–6.711)	0.313			
The number of recurrent lesions (1/ 2–3)	2.660	(1.042–6.792)	0.041*	1.989	(0.634–6.242)	0.239
Post-operative laboratory data						
Lymphocytes to Monocytes ratio (Low/ High)	0.791	(0.282–2.221)	0.656			
Neutrophils ato Lymphocytes ratio (Low/ High)	1.331	(0.474–3.740)	0.588			
Platelet to Lymphocytes ratio (Low/ High)	1.055	(0.416–2.680)	0.911			
Total Lymphocytes count (Low/ High)	1.883	(0.670–5.293)	0.230			
PD-L1 expression						
< 1%/ > 1%	1.242	(0.451–3.422)	0.675			
< 5%/ > 5%	0.647	(0.184–2.280)	0.498			
< 10%/ > 10%	0.980	(0.222–4.325)	0.978			
CD103 expression (Low/ High)	0.330	(0.119–0.915)	0.033*	0.263	(0.081–0.849)	0.026*

*CI* Confidence Interval, *scc* squamous cell carcinoma, *irAE* immune-related Adverse Event

^†^Cox proportional hazard model; *p < 0.05, statistically significant

^a^TNM Classification of Esophageal squamous cell carcinoma, 8th ed

### Correlation between PD-L1 and CD103 expression within tumor sites

**Fig. 3:**
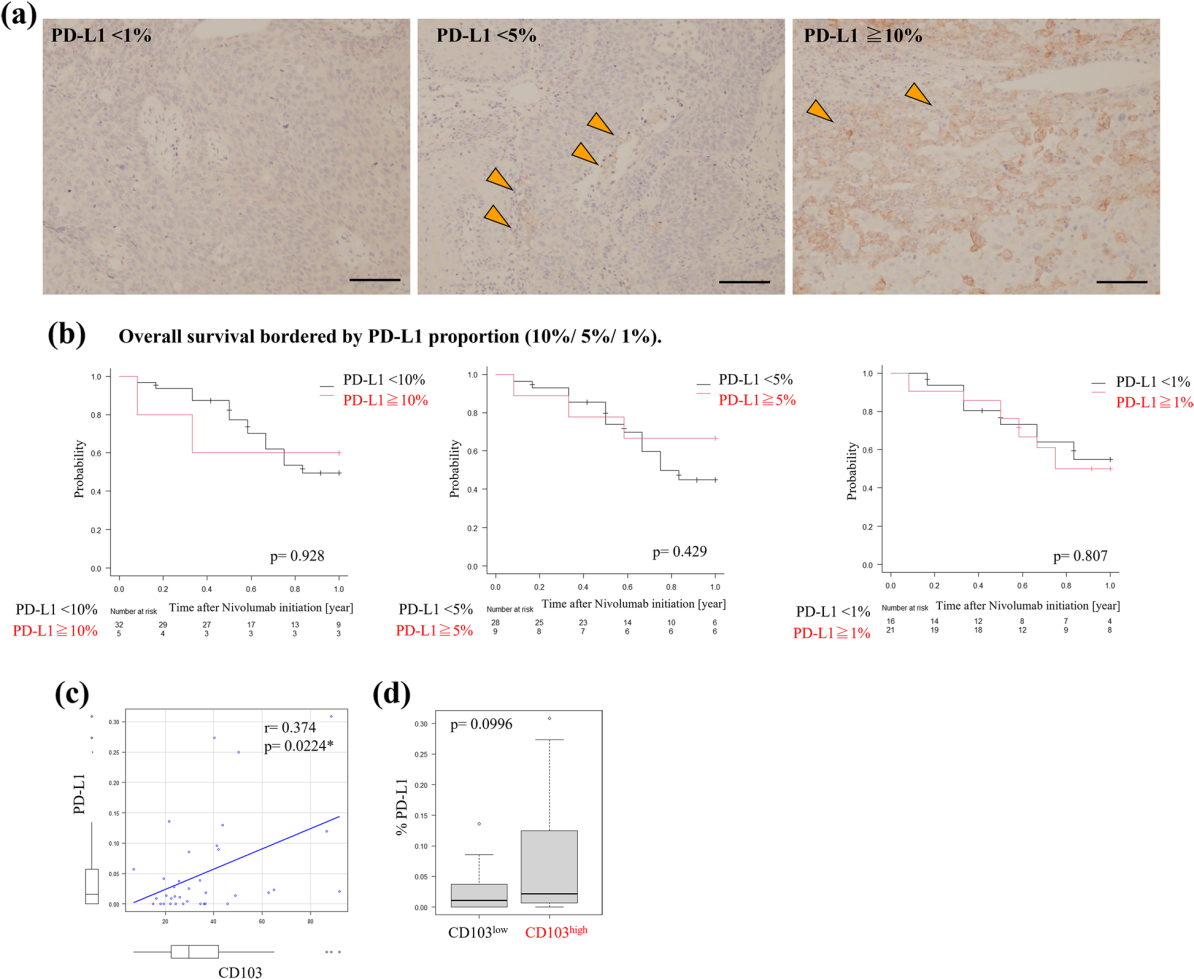
**a** Representative images of immunohistochemistry using anti-PD-L1 antibody. Scale bars 100 mm. Those shows various PD-L1 expression within tumors (arrow heads). **b** We set some borderlines of PD-L1 expression (1%, 5%, 10%), and compared OS based on each value using Kaplan Meyer plots and the log-rank test. *p < 0.05, statistically significant. **c** We compared PD-L1 and CD103 expression within tumors using Spearman’s rank correlation coefficient. **d** Box plot. We compared the percentage of PD-L1 expression within tumors between CD103^high^ and CD103^low^ groups

We performed immunohistochemistry on the primary tumors using anti-PD-L1 antibody and then analyzed the correlation between PD-L1 and CD103 expression. Similar to the ESCC clinical trial [2], we classified PD-L1 expression (1%, 5%, and 10%) (Fig. 3a). Based on each border, we compared OS with PD-L1 (Fig. 3b). However, PD-L1 expression did not significantly prolong survival in this study. Immunostaining showed that only the CD103^high^ patients had high PD-L1 expression, whereas most had low PD-L1 expression. We examined the correlation between CD103 and PD-L1 expression using Spearman’s rank correlation coefficient and found that the CD103 expression was weakly correlated with PD-L1 expression (*r* = 0.374, *p* = 0.022; Fig. 3c). In addition, the results of the Mann–Whitney *U* test revealed that there was a trend of higher PD-L1 expression in CD103^high^ than in CD103^low^ without a statistical difference (Fig. 3d).

### Infiltration of T_RM_ precursors

**Fig. 4:**
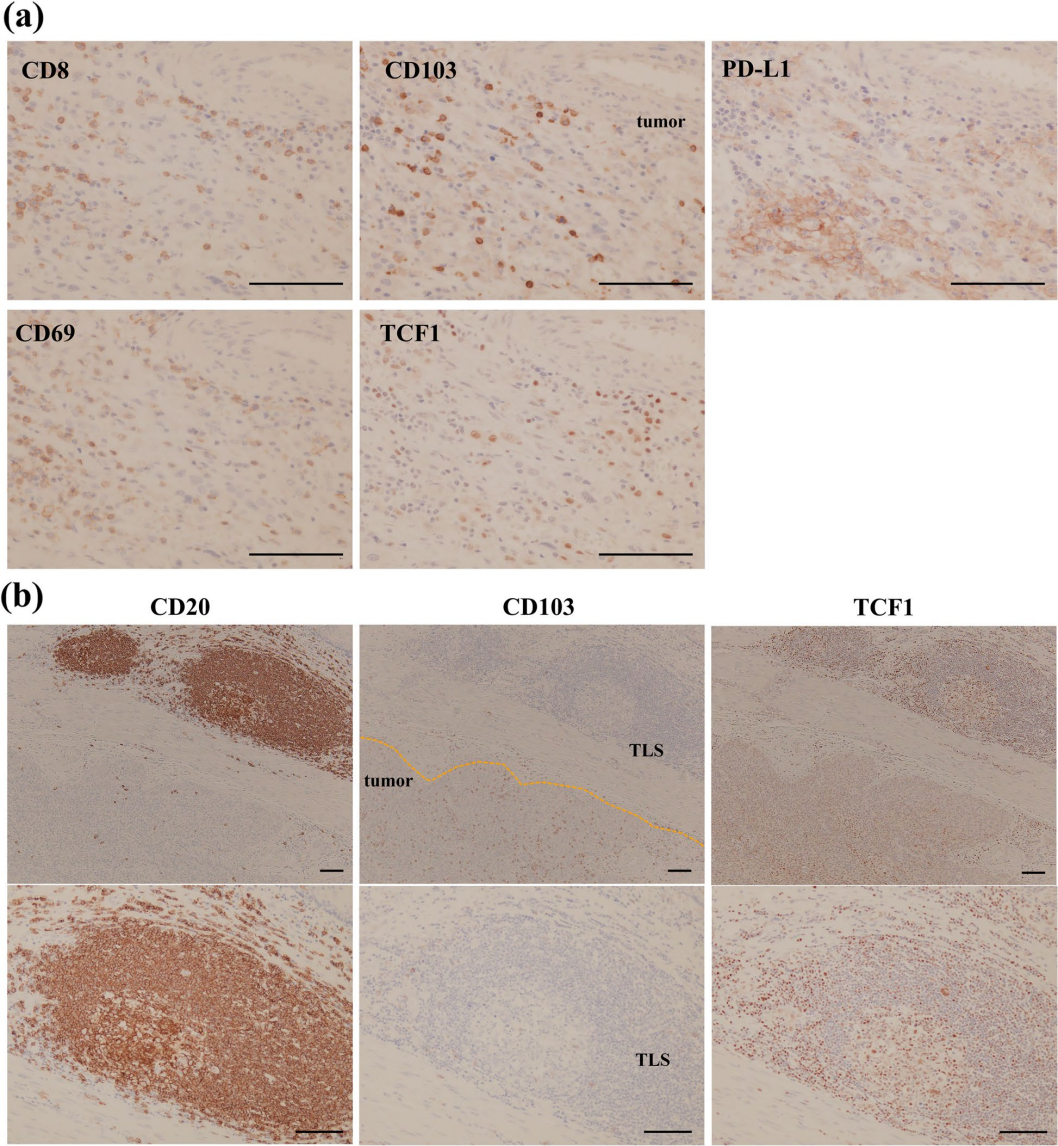
**a** Representative images of immunohistochemical analyses using anti-CD8, anti-CD103, anti-PD-L1, anti-CD69, and anti-TCF1 antibodies. Scale bars 100 mm. **b** Representative images of immunohistochemistry using anti-CD20, anti-CD103, and anti-TCF1 antibodies. Scale bars 100 mm

We confirmed that most CD103^+^ infiltrates co-expressed CD8 by immunohistochemistry, such as in our previous report [6] (Fig. 4a). ICIs are reported that they revitalize CD8^+^ TILs which are pre-exhausted status [16–18]. It was implied that T_RM_ infiltration is associated with pre-exhausted lymphocytes, T_RM_ precursors. We additionally examined the expression of CD69 and TCF1 because CD69 is one of the representative T_RM_ markers, and both CD69 and TCF1 is possibly expressed on T_RM_ precursors [9] (Fig. 4a). However, CD69^+^ and TCF1^+^ cells did not always correspond to their distribution to CD103^+^ infiltrates. Both molecules are relatively expressed in naïve or immature cells. We also immunohistochemically stained tertiary lymphoid structures (TLSs) adjacent to primary tumors using anti-CD20 antibody that form peripherally and are structurally and functionally similar to secondary lymph organs. TLSs surrounded numerous TCF1^+^ cells (Fig. 4b).

### Association between CD103^+^ infiltrates and the laboratory data

**Fig. 5:**
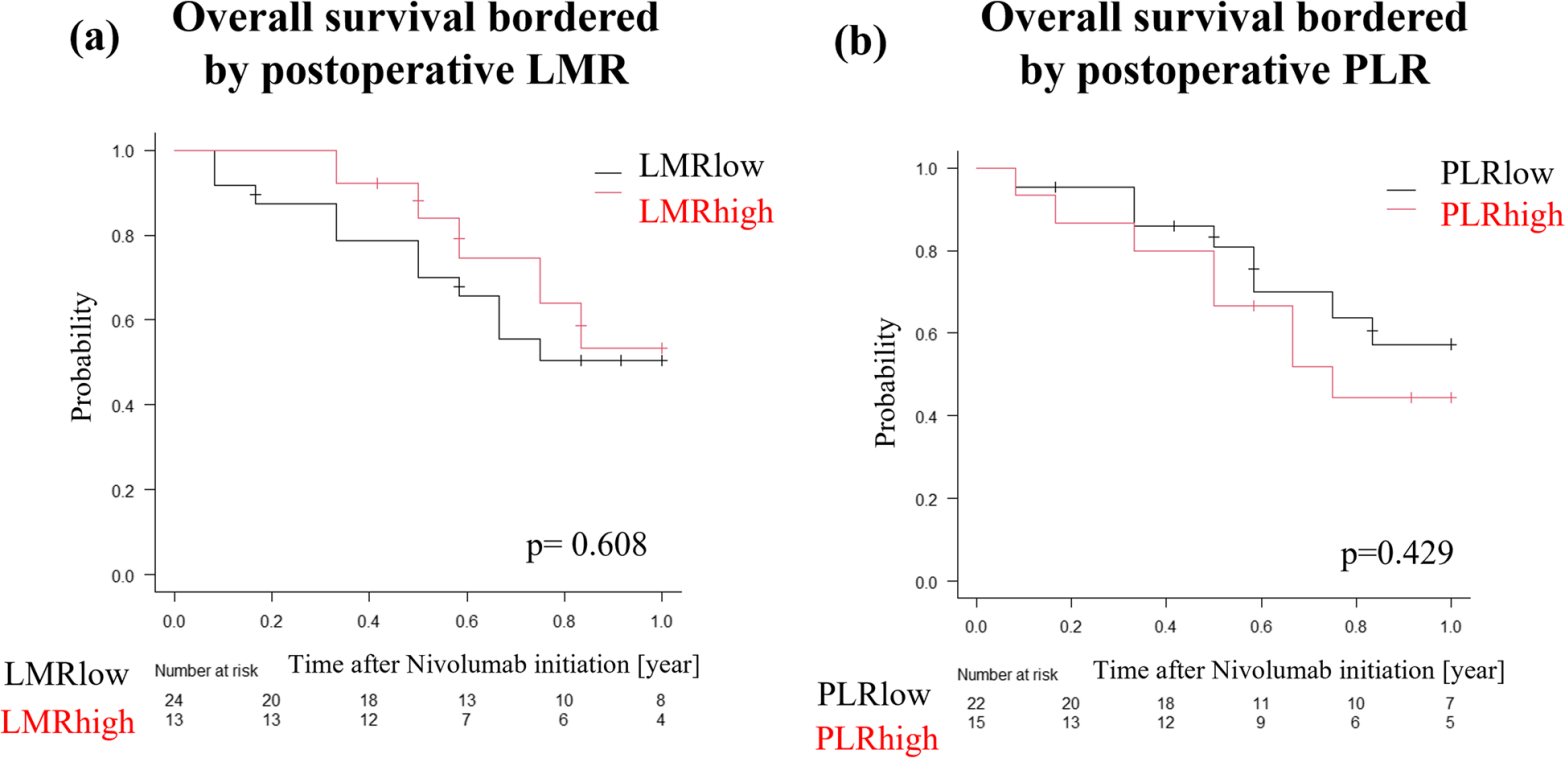
**a**, **b** Comparison of OS using Kaplan-Meyer plots and the log-rank test. *p < 0.05, statistically significant. **a** Comparison between postoperative LMR high and low. **b** Comparison between postoperative PLR high and low

We examined the association between CD103 expression and laboratory data perioperatively and before nivolumab initiation (Supplementary Table 1). We found that postoperative LMR was weakly correlated (*r* = 0.386, *p* = 0.018), and the postoperative PLR was inversely correlated (*r* = -0.340, *p* = 0.040) with CD103^+^ infiltrates. In contrast, there was no correlation between CD103 expression and the parameters before surgery or nivolumab initiation. We then set cut-off values for postoperative LMR and PLR using the ROC curve (Supplementary Fig. 1b, c). Based on the cut-off values, we divided the patients into two groups, high and low. However, there were no significant differences in OS between high and low levels (Fig. 5a, b).

## Discussion

This study found that CD103^+^ cells were an evaluable biomarker for ICIs treatment as second-line chemotherapy for recurrent esophageal cancer. Nivolumab is a commonly used immune checkpoint inhibitor in Japan [1]. It is thought to be highly effective in patients with tumors highly expressing PD-L1 or including high immunogenicity, the so called ‘hot tumor’ [19]. We refer to PD-L1 expression as an efficacy indicator of ICIs in various types of tumors [3, 20–23]. In ESCC, patients with high PD-L1 levels exhibit favorable effects from immunotherapy, including PD-1 blockade plus chemotherapy [2, 24–26]. Evaluation methods for PD-L1 expression include TPS or combined positive score (CPS) [27, 28]. TPS is calculated by PD-L1 expressed tumor cells, whereas CPS has PD-L1-positive immune cells, such as lymphocytes or macrophages. We adopted TPS in this study based on the clinical trial [2]. TPS cannot be differentiated between responders and non-responders to nivolumab monotherapy in some malignancies [29, 30]; however, PD-L1 positivity has a modest survival benefit after nivolumab administration in ESCC [2]. However, our study did not indicate that PD-L1 expression is advantageous for nivolumab therapy. In addition, PD-L1 assessment is time-consuming and requires expert technicians. Moreover, we cannot ignore the heterogeneity of PD-L1^+^ cells at tumor sites.

We previously reported that the infiltration into ESCC of CD103^+^ T_RM_, which have potent cytotoxicity, prolongs survival [6]. In particular, in lymph node metastatic ESCC cases, T_RM_-rich metastatic portions provide a better prognosis than T_RM_-poor portions [6]. These results suggest that the presence of T_RM_ at tumor sites reflects the function of host immune mechanisms [6]. In this study, CD103^high^ exhibited a better response to nivolumab treatment and a better prognosis than CD103^low^. Notably, in CD103^high^ and CD103^low^ patients, the 12-month OS rate was 70.8% and 37.2%, respectively, and the 12-month PFS rate was 31.2% and 0.00%, respectively. In neoadjuvant chemotherapy, the poor response group (grade 0-1b) was significantly different in the number of CD103^+^ cells from the good response group [2, 3]. This result suggested that tumor shrinkage by chemotherapy makes CD103^+^ cell detection decrease. Even the poor response group to chemotherapy can possibly get a good response to ICIs if T_RM_ richly infiltrates. CD103^high^ also contributed to the improvement in DOR, DCR and ORR; many patients in the high group could have continued following treatments even if they discontinued nivolumab treatment. We found that the BOR to nivolumab gave no significant differences in the number of CD103^+^ cells. However, CD103 expression was significantly associated with OS and PFS. Although the BOR to nivolumab didn’t significantly differ between the two groups, statistical differences were observed in OS and PFS after nivolumab initiation. The effect of nivolumab monotherapy develops a bit longer time after first administration compared to chemotherapy: therefore, the BOR to nivolumab between the two groups might be comparable. However, as reported, nivolumab binds on T cells for more than 20 weeks after last administration [31]. In addition, subsequent chemotherapy following nivolumab, primarily taxanes, also contributes to improvement of survival. That is because treatments after nivolumab discontinuation reduce regulatory T cells and other immune suppressive cells, leading to revitalizing more T_RM_ by the residual efficacy of preceding nivolumab [32]. According to our previous report on gastric cancer, CD103^+^ TILs were inferred to cause a massive improvement in response to nivolumab treatment [33]. Therefore, CD103^+^ TILs, called T_RM,_ may play a crucial role in response to ICIs. IrAEs caused by ICIs treatment are reportedly associated with good response to ICIs treatment [34]. Our results also showed that CD103^high^ patients experienced more irAEs. Recently, PD-L1 positivity were associated with CD103^+^ T_RM_ rather than CD8^+^ TILs [17]. We clarified the correlation between CD103 and PD-L1 expression; however, CD103^+^ TILs responded more sensitively to nivolumab therapy than PD-L1 positivity. We expect T_RM_ to be a simple and promising surrogate marker for ICIs.

Tumor antigen chronically stimulates and makes T cells exhausted. ICIs bind and revitalize pre-exhausted CD8^+^ T cells if T cells are not terminally exhausted [16–18]. In particular, T_RM_ has a strong cytotoxicity and resides in the tumor sites resulting in much expressing PD-1. Therefore, T_RM_ may tend to benefit from ICIs and become a promising surrogate marker for ICIs. T_RM_ precursors have been indicated to have a pre-exhausted status and high potential for antitumor immunity [9, 35–37]; we applied immunostaining to TCF1^+^ and CD69^+^ cells, which are thought to be characteristic markers of T_RM_ precursors. In particular, TCF1 expression is related to T cell exhaustion; TCF1^+^ T cells are named progenitor-exhausted T cells, whereas TCF^−^ counterparts are called terminal-exhausted T cells [18, 38]. Baharom et al. reported that these progenitor-exhausted T cells induce a superior antitumor response to ICIs [39]. In the present study, we performed immunostaining on them to examine the distribution and direct association between ICIs treatment and these factors. However, these markers were not always present in CD103^+^ TILs, and we could not detect differences in their expression between CD103^+^ high and low infiltrates. In contrast, TCF1^+^ cells existed around the TLSs. TLSs are formed peripherally near tumors, such as secondary lymph organs and aggregates of various immune cells around B cells, and are associated with antitumor immunity and a good prognosis [40, 41]. T_RM_ are associated with TLS formation by CXCL13 [42], and our group has reported a relationship between TLS and T_RM_ [43]. TLSs have been reported to facilitate the response to ICIs [44–47], possibly due to the interaction of antitumor immunity with T_RM_. Our immunostaining implied that there is reciprocity between TLS and T_RM_ through TCF1^+^ lymphocytes; therefore, further investigations would help discover novel targets for ICIs treatment.

In addition, we compared the survival prognosis between blood test parameters and CD103 expression. Various reports have mentioned that blood cell markers including NLR, LMR, and PLR are promising response indicators to ICIs [13–15, 48]. Whether TILs in the surgical specimens were related to these markers was scarcely noted. In this study, CD103 expression within primary tumors correlated significantly with postoperative parameters than with preoperative parameters or parameters before nivolumab initiation. We speculate that there are two reasons for this finding. First, because tissue samples were obtained from surgical specimens, the condition of the tumor specimens might closely resemble the perioperative parameters. Second, most ESCC patients received chemotherapy just before surgery or nivolumab administration, causing laboratory data to be affected by the prior chemotherapy. However, these laboratory data did not indicate a response to nivolumab therapy or any survival benefits. Based on our results, TILs infiltration may not be commensurate with laboratory data in predicting the effectiveness of ICIs.

This study had several limitations. First, the sample size was small, and the duration of observation was short. That is because usefulness of T_RM_ evaluation in biopsy tissues has been unclear in assessing unresectable advanced esophageal cancer, and there were not many patients with recurrent esophageal cancer who received nivolumab yet. Therefore, more extensive and long-term studies are required. Second, this study primarily employed immunohistochemistry. Consequently, we could not demonstrate the actual mechanism of T_RM_ to nivolumab treatment. It would be helpful to investigate the differentiation and function of T_RM_ related to ICI treatment in greater detail using other approaches.

## Conclusions

In conclusion, CD103^high^ infiltrates were associated with a favorable prognosis after nivolumab initiation. CD103^+^ TILs, T_RM_, were examined simply and sensitively to determine the response to ICIs. Therefore, T_RM_ are instrumental in antitumor immunity and are probably a significant surrogate marker for the response to ICIs.

### Supplementary Information

**Additional file 1: Supplementary Table 1.** Details of laboratory parameters and Correlations of CD103 infiltrates with laboratory.

**Additional file 2. **

## Data Availability

The datasets used and analyzed in this study are available from the corresponding author upon reasonable request, and most of the original data are included within the article.

## Abbreviations

ESCC: Esophageal squamous cell carcinoma
ICI: Immune checkpoint inhibitor
PD-1: Programmed death 1
PD-L1: Programmed death ligand 1
TIL: Tumor-infiltrating lymphocyte
T_RM_: Tissue-resident memory T cell
FP: 5-Fluorouracil + cisplatin
FGP: 5-Fluorouracil + nedaplatin
DCF: Docetaxel + cisplatin + 5-fluorouracil
ROC: Receiver operating characteristic
PFS: Progression-free survival
TPS: Tumor proportional score
LMR: Lymphocytes to monocytes ratio
TLC: Total lymphocytes count
NLR: Neutrophils to lymphocytes ratio
PLR: Platelets to lymphocytes ratio
BOR: Best overall survival
ORR: Objective response rate
CR: Complete response
PR: Partial response
DCR: Disease control rate
SD: Stable disease
DOR: Duration of response
OS: Overall survival
irAE: Immune-related adverse event
TLS: Tertiary lymphoid structure
CPS: Combined positive score
