# Supplementary figures and images for: Significance of CD103+ tissue-resident memory T cells for predicting the effectiveness of immune checkpoint inhibitors in esophageal cancer
Source: BMC Cancer. 2023 Oct 20;23:1011. doi: 10.1186/s12885-023-11438-5 (PMC10588150; doi:10.1186/s12885-023-11438-5)

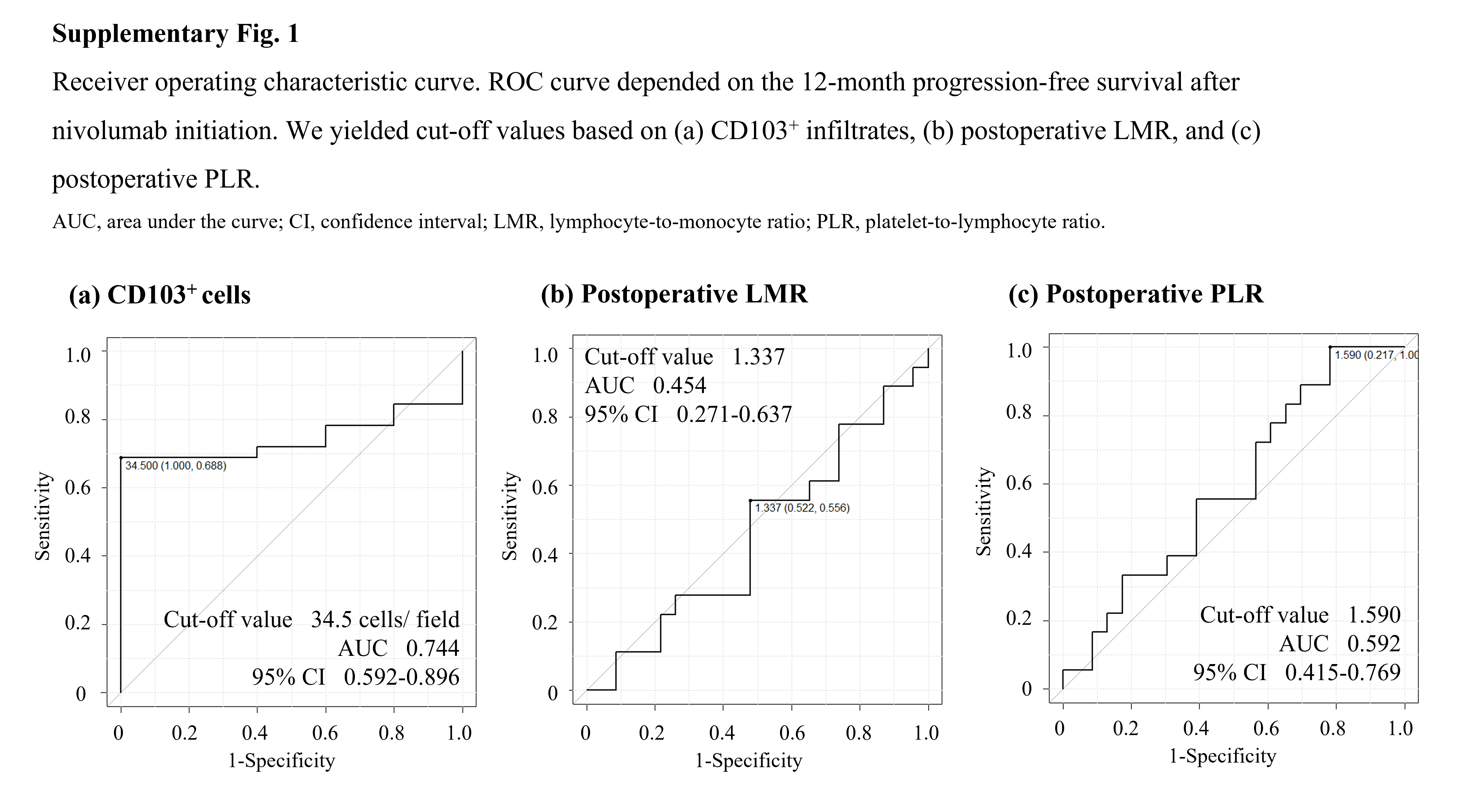

Supplement: Supplementary file 2 — Additional file 2. [file 12885_2023_11438_MOESM2_ESM.tif]
